# Predictors of Sun Protective Behaviors among Latino Day Laborers

**DOI:** 10.1155/2018/3454309

**Published:** 2018-01-28

**Authors:** Javier F. Boyas, Vinayak K. Nahar

**Affiliations:** ^1^Department of Social Work, School of Applied Sciences, University of Mississippi, 214 Longstreet Hall, P.O. Box 1848, Oxford, MS 38677, USA; ^2^Center for Animal and Human Health in Appalachia, College of Veterinary Medicine, DeBusk College of Osteopathic Medicine, and School of Mathematics and Sciences, Lincoln Memorial University, 6965 Cumberland Gap Parkway, Harrogate, TN 37752, USA; ^3^Department of Dermatology, School of Medicine, University of Mississippi Medical Center, 2500 N. State St., Jackson, MS 39216, USA

## Abstract

**Objectives:**

Despite the substantial solar ultraviolet radiation experienced by Latino day laborers, little attention has been given to factors that are associated with sun protective behaviors. The purpose of this study was to examine psychological and nonpsychological predictors of sun protective behaviors among Latino day laborers.

**Methods:**

This cross-sectional study included a nonrandom sample of 137 Latino day laborers recruited from Mississippi and Illinois. Participants completed a self-report survey instrument, available in English and Spanish, on sun protective behaviors.

**Results:**

Multivariate regression results showed that sun protective behaviors were significantly greater among Latino day laborers: (a) who had greater perceptions that their supervisor also engaged in sun protective behaviors (*β* = 0.25, *p* ≤ 0.01); (b) who reported higher levels of health literacy (*β* = 0.23, *p* ≤ 0.001); (c) who have greater knowledge of skin cancer risk factors (*β* = 0.21, *p* ≤ 0.01); and (d) who have skin tone that was self-perceived to be more prone to sunburns (*β* = 0.19, *p* ≤ 0.01).

**Conclusions:**

Latino day laborers possess marginal levels of skin cancer knowledge and engage minimally in sun protective behaviors. Skin cancer prevention interventions are warranted for this high-risk group, particularly in the locations in which Latino day laborers work.

## 1. Introduction

The phenomenon of day labor in the United States (U.S.) can be traced back as early as the 1800s in New York City, where unemployed men and women would assemble in the street markets in hopes of gaining employment [1]. Today, day laborers are part of an informal labor force that account for roughly 8.3 million of the unauthorized workers in the U.S. [2]. The only national study conducted in the U.S. on day laborers estimated that roughly 117,600 workers seek out employment as day laborers on a daily basis [3].

It is well established that the majority of the day laborer work force is made up of Latinos and that Latino day laborers (LDLs) are often employed in industries that include a number of occupational hazards [3]. Some of these workplace threats include unsafe mechanized tools and equipment, environments with high noise levels, exposure to dangerous chemicals, work at risky heights, lack of personal protective equipment, and little or no safety oversight [4–8]. Although these workplace hazards have been acknowledged and studied, the regular exposure of LDLs to solar ultraviolet radiation (UVR) during peak periods has remained unexplored [9], despite frequent employment in trades that take place outdoors, such as landscaping, gardening, roofing, and construction [5, 8]. Daily UVR exposure in excess is considered an occupational health and safety issue that places outdoor workers at risk of developing melanoma and other nonmelanoma forms of skin cancer [10, 11], which is alarming given that, in one study, 93.1% of LDLs reported being exposed to too much sun [12].

Skin cancer is by far the most commonly diagnosed cancer in the U.S. [13]. Roughly 5.4 million cases of nonmelanoma skin cancer are diagnosed every year [14]. According to recent estimates, about 87,110 new cases of melanoma will be diagnosed in 2017 and about 9,730 people are expected to die from this type of cancer [13]. Among the broader Latino population, incidence rates of melanoma are also on the rise; however, this upward trend seems to only be in certain geographic locations of the U.S. [15]. This rise is challenging for Latinos given that only one in fourteen Latinos reports ever having a skin examination by a physician [16]; only 3.2% have been trained on how to perform a skin self-examination [17], and they are less likely to wear sun protective clothing or apply sunscreen with a protection factor of 15 or higher [17]. As a result, compared to non-Hispanic whites, Latinos are more likely to be diagnosed with melanoma in advanced stages that often result in poorer cancer survival rates [18, 19].

Adopting sun protective behaviors (SPBs) has been a mediating primary prevention strategy to help reduce harmful UVR effects [20, 21]. Primary prevention practices include avoiding direct sun exposure between 10 am and 4 pm, using wide-brimmed hats, wearing long-sleeve shirts, wearing sunglasses, wearing gloves and other protective clothing to block direct sun exposure, and using sunscreen with sun protection factor (SPF) of 30 or higher [22]. Although SPBs have been identified as protective factors in the literature, Latinos have not adopted these behaviors with any consistency [10, 23–25]. For example, in a 2015 study of Latino outdoor workers, 42.9% of participants reported never using sunscreen when working, and another 26.1 reported rarely using sunscreen while working [10]. Since there is no information currently on LDLs and SPBs, the existing literature among the broader Latino population and Latino outdoor workers was reviewed to determine what factors correlated with SPBs. The current literature suggests that use of SPBs is likely to be associated with sociodemographic characteristics [10, 25], acculturation levels [10, 23], knowledge of skin cancer risk factors [25], workplace norms that are associated with how supervisors and coworkers support use of SPBs [26], hours of outdoor work [10], and psychosocial factors, such as perceived barriers and perceived risk based on skin tone [25, 26].

The analysis also examined association between self-efficacy and SPBs. Self-efficacy is the confidence that an individual has in his/her ability to perform a certain behavior [27, 28]. Little is known as to whether self-efficacy contributes to SPBs among LDLs. Moreover, the present study assessed whether health literacy correlates with SPBs. Health literacy is an important aspect of health to consider since low levels have been shown to have adverse effects on several health outcomes [29, 30]. This issue may be much more germane to Latinos, who consistently report possessing marginal levels of health literacy [31–33]. One study revealed that Latinos who were limited-English proficient reported the second lowest levels of health literacy, compared to other racial and ethnic groups [33].

Despite the heightened levels of exposure to UV rays experienced by LDLs, minimal research has been carried out that examines the extent to which LDLs use sun protective practices. One study focused on examining use of sunscreen practices among Latino outdoor workers [10]. However, that study did not explore the couse of various sun safe strategies, nor did it examine perceived barriers to SPBs. To date, there are no published studies that assess LDLs' sun protective perceptions and practices. The purpose of this study was to examine predictors of SPBs among LDLs. The present study examined SPBs among a nonprobability sample of self-identified LDLs residing in a southern state and Midwestern city. The Latino population in the southern U.S. has grown substantially in the past 25 years [34], yet, very little research exists that documents the contextual experience of SPBs among LDLs in this region of the U.S. Based on the broader literature, it was hypothesized that increased levels of SPBs are predicted by LDLS with higher levels of achieved education, higher incomes, fairer skin tone, moles on their skin, higher levels of acculturation, more knowledge of risk factors, workplace norms that support increased use of sun protective practices, lower perceived barriers, higher levels of self-efficacy, and higher levels of health literacy. Understanding the role of the aforementioned psychological and nonpsychological correlates may identify specific areas to target and guide future interventions aimed at improving use of SPBs among LDLs.

## 2. Materials and Methods

### 2.1. Study Design and Participants

The cross-sectional survey used in the present study was carried out between July and November of 2014 with Hispanic/Latino day laborers residing in either Mississippi or Illinois. This time frame allowed us to assess the summer sun protective behaviors in order to avoid a possible recall bias. In Mississippi, data were collected until November. In the South, there are more extended periods of sunshine and warm temperatures that carry over well into the fall season. However, in Illinois, data collection stopped in early October, when the number of sunny and warm days becomes fewer. Recruitment and data collection procedures have been reported elsewhere [9]. The sample for this study was comprised of a community-based nonrandom sample. Participants were included if (1) they self-identified as either Hispanic or Latino, (2) they were aged 18 years or older, (3) they actively sought informal and short-term temporary employment, (4) they had no cognitive impairment, (5) they were not institutionalized, and (6) they are resided in Mississippi or Illinois. A self-administered questionnaire, available in Spanish and English, took approximately 55 minutes to complete. All participants were also provided an option of having the survey read to them to make sure that potential participants with literacy issues were not discouraged from participating. In Illinois, roughly 40% of LDLs that were approached to take part in the study declined to participate, whereas 30% of LDLs declined in Mississippi. All members of the research team were bilingual (English-Spanish). To establish the reliability of data collection, the research assistants were trained on appropriate interviewing skills and were asked to familiarize themselves with each item of the survey. A research honorarium of $20.00 was given to participants. Written informed consent was obtained from all participants. The Institutional Review Board at the University of Mississippi granted ethical approval.

### 2.2. Measures

The survey instrument used in the current study was predominantly developed using existing validated instruments [35–42]. Spanish versions were available for some of these instruments [37]. If a Spanish version of the instrument was not available, the research team of two Mexican Americans, a Peruvian, and a Venezuelan researcher carried out the translation. During the translation process, the goal was to make the survey comprehensible to individuals with low levels of education. The translation generated a survey with a Flesch-Kincaid Readability level of 5.7 [43].

#### 2.2.1. Dependent Variable

The SPBs index consisted of nine single items that were used for assessing use of SPBs (e.g., wide-brimmed hat, long-sleeved shirt, shirt with collar, limiting midday sun, sunglasses, sunscreen, gloves, and covering head and face) [37]. A sample item was the following: “During the summer months at work, how often do you wear a hat with a surrounding brim of at least 2.5 inches when you are in the sun for more than 15 minutes?” Each item was measured on a 5-point Likert scale with responses ranging from 1 (never) to 5 (always). These nine items were summed and higher scores indicated higher use of SPBs. In the current study, the internal consistency for this scale was Cronbach's *α* = 0.85.

#### 2.2.2. Predictor Variables

The following sociodemographic background information was gathered in this study. Age was a single, continuous measure. Education was an ordinal measure that was dichotomized in the analyses (1 = high school completed and beyond; 0 = less than high school completed). Foreign-born day laborers were asked a single question as to whether they were educated in their native country (1 = yes; 0 = no). Annual income was a single, ordinal variable that was dichotomized in the analyses (1 = earned more than $20,000; 0 = earned less than $20,000). Immigration status in U.S. was a nominal measure that was dichotomized in the analyses (1 = legally in the U.S.; 0 = undocumented status). Health insurance coverage was a single, dichotomous variable (1 = has health insurance coverage; 0 = no health insurance coverage). The number of years living in U.S. was a continuous, single variable. The participants were asked if they had any moles on their body. This was a single, dichotomous variable (1 = yes, 0 = no). Perceived skin type was a single, ordinal measure that was collapsed into a dichotomous variable (1 = always burn, never tans, usually burn, tans with difficulty, and sometimes mild burn, gradually tans to a light brown; 0 = rarely burn, tan with ease to moderate brown, very rarely burns, tans very easily, and never burns, tans very easily, deeply pigmented).

#### 2.2.3. Acculturation

The Short Acculturation Scale for Hispanics for use with the Latino population was used to determine acculturation levels [35]. Respondents were asked about what language they read and speak, what language they usually spoke at home, in which language they usually think, and what language did they speak with their friends. These questions were answered on a 5-point Likert-type scale, including the following categories: 1 = (only Spanish) to 5 = (only English). In the current study, the internal consistency for this scale was Cronbach's *α* = 0.91, with higher scores representing higher levels of acculturation.

#### 2.2.4. Knowledge of Cancer Risk Factors

Seven items from knowledge scale developed by Cottrell and colleagues were used to measure participants' knowledge about risk factors associated with skin cancer [37]. A sample item was the following: “Which of the following are increased risk factors related to skin cancer? Having dark colored skin?” The responses for each item were measured on a “yes” (= 1), “no” (=0), and “do not know” (=0), scale. If the respondent answered “do not know,” the answer was marked as a “zero.” After summing the items, the total possible score for knowledge ranged from 0 to 7, with a higher score indicating higher knowledge of risk factors of skin cancer.

#### 2.2.5. Workplace Norms

Work place norms were assessed by two single items that were developed by the authors of the current study. The two single items were as follows: (a) How much do you think your supervisor support engaging in sun protective behaviors at work? (b) How much do you think your coworkers support engaging in sun protective behaviors at work? Both items were measured on a 5-point Likert scale with responses ranging from 1 (strongly oppose) to 5 (strongly support).

#### 2.2.6. Time Spent Outdoors at Work between 10 a.m. and 4 p.m.

A single item was used to measure time spent outdoors at work during peak sun times. This was a continuous measure that asked respondents to state how long they were in the sun while at work between 10 a.m. and 4 p.m.

#### 2.2.7. Perceived Barriers

Perceived barriers for not practicing sun protection were assessed by 12 Likert-type items, ranging from 1 (strongly agree) to 5 (strongly disagree) [38, 39]: for example, “I often forget to protect myself from the sun,” “Sun protection measures are expensive,” and “It is not always convenient to protect myself from the sun.” After summing the items to create a scale, the total possible score for perceived barriers ranged from 13 to 65, with a higher score denoting greater perceived barriers towards use of sun protection methods. In the current study, the internal consistency for this scale was Cronbach's *α* = 0.71.

#### 2.2.8. Self-Efficacy in Relation to SPBs

A total of seven items were used for assessment of self-efficacy level to engage into SPBs (e.g., seeking shade, wide-brimmed hat, long-sleeved shirt, long pants, gloves, sunglasses, and sunscreen) [40, 41]. A sample item was the following: “How confident are you to wear a long-sleeved shirt when you are in the sun for more than 15 minutes between 10 AM and 4 PM?” The response categories ranged from 1 (“not at all confident”) to 10 (“highly confident and certainly can do”). These seven items were summed and the total possible score for self-efficacy section ranged from 10 to 70, with a higher indicating score higher confidence level in being able to protect oneself when in the sun for more than 15 minutes between 10 a.m. and 4 p.m. In the current study, the internal consistency for this scale was Cronbach's *α* = 0.88.

#### 2.2.9. Health Literacy

Health literacy of the participants was measured using three brief screening questions developed by Chew and colleagues (2004) [42]: for example, “How confident are you filling out medical forms by yourself?” These items were answered on a 5-point Likert-type scale, including the following categories: 1 = not at all confident to 5 very confident. Items were summed to create the health literacy scale. In earlier studies that included Latino respondents, this scale produced adequate levels of internal consistency, Cronbach's *α* = 0.73 [31]. In the current study, the internal consistency for this scale was Cronbach's *α* = 0.83, with higher scores representing higher levels of health literacy.

### 2.3. Analytic Strategy

Descriptive statistics were generated to distinguish the study sample in terms of sociodemographic characteristics. Bivariate analyses were computed by way of Pearson's *r* zero-order correlations to determine the direction and size of the relationship between all predictor variables and SPBs. The main analysis employed multiple linear regression to determine if a significant relationship exists between SPBs and predictor variables. All variables were entered into the model concurrently since it was not hypothesized a priori the relative importance of each predictor variable and its relation to SPBs. To construct the multivariate model, only variables which were statistically significant in the correlation analyses were included. The results in the multivariate model were presented by the size of the standardized beta. Regression diagnostics were examined to confirm that core assumptions were not violated [44]. The assumptions of normality, linearity, and homoscedasticity of the residuals were examined using normal probability plots and scatter plots. The variance inflation factor (VIF) was used to test for multicollinearity. Multicollinearity was considered a concern if the VIF coefficient was >10. After each assumption was assessed, no violations were identified. Statistical significance was measured at the 95% confidence interval level (*p* ≤ 0.05). All statistical analyses were conducted using IBM SPSS version 22.0 [45].

## 3. Results

A total of 138 Hispanic/Latino day laborers participated in this study. Of the 138 participants, 55.8% (*n* = 77) were from Mississippi and 44.2% (*n* = 61) were from Illinois. One of the study participants was a female who was excluded from all data analyses.  Table 1 describes the study's sample and related variables. The sample consisted of 72% self-identifying as being of Mexican ancestry. The average age was 35.40 (SD = 9.989). Education levels were modest; 44.5% reported having less than an elementary school education. Sixty-nine percent of respondents reported earning less than $20,000 in yearly income. Thirty-seven percent of the men reported being undocumented, noncitizens or permanent legal residents. The average LDL had been in the U.S. an average of 11.15 (SD = 9.48) years. The sample could be characterized as having lower levels of acculturation. The majority of respondents worked in the areas of construction (41.6%) and landscaping (18.2%). The respondents had low levels of knowledge of skin cancer risk factors; they were only able to successfully identify, on average, 2.6 out of 6 risk factors. Most of the respondents reported that they rarely burn and tan with ease (33.3%).

Data were extracted by region to determine if statistical differences existed between the data collected in Mississippi and Illinois; however, no significant differences were identified on any of the sun protective perceptions or behavior variables. Two areas where the data did differ significantly were in the areas of legal status and acculturation levels. A higher number of LDLs reported being undocumented in Mississippi compared to Illinois (*x*^2^ = 13.084, *p* ≤ 0.05). Acculturation levels of LDLs were significantly higher in Illinois (*M* = 6.65, SD = 3.13), compared to Mississippi (*M* = 5.65, SD = 2.71).

Overall, LDLs reported very modest use of SPBs (*μ* = 16.23, SD = 5.11). The most commonly reported behaviors were use of wearing something over their head, such as a hat, cap, or visor (*μ* = 2.82, SD = 1.07) and wearing sunglasses (*μ* = 2.13, SD = 1.08). A major concern was found among the least reported behaviors, which were wearing any protective gear over the face (*μ* = 1.29, SD = 0.54), wearing a long-sleeved shirt (*μ* = 1.58, SD = 0.76), and wearing sunscreen with SPF of 15 or higher (*μ* = 1.65, SD = 0.94).

Among LDLs, several factors were significantly correlated with SPBs (see Table 2). Results of the zero-order correlation suggest that SPBs were significantly correlated with acculturation level, income, legal status, skin type, knowledge, length of time in the U.S. for those who were foreign-born, health literacy, barriers, and SPBs of supervisors and coworkers. LDLs: with higher income, who are not legal citizens, naturalized, or residents, with skin types more prone to sunburns, who have resided in the U.S. a longer period of time, and who were more acculturated reported significantly more SPBs. Increased knowledge of skin cancer risk factors and higher levels of health literacy were also significantly associated with SPBs. LDLs who reported more confidence in using sun protective methods and less barriers to using sun protective methods also reported significantly more increased SPBs. LDLs also reported significantly higher SPBs when they had greater perceptions that sun protection measures were being taken by their supervisors and coworkers. The strongest correlation of SPBs was shared with higher perceptions that sun protection measures were being taken by their supervisors (*r* = 0.61, *p* ≤ 0.01). Age, education, education in native country, health insurance coverage, having moles, and time spent working outdoors during peak times did not significantly relate to SPBs.

The multivariate results suggest that several factors significantly predicted SPBs (see Table 3). The model was significant (*F* = 20.658, *p* = 0.001). The predictors in the model accounted for 62% of the variance in SPBs. Among LDLs, SPBs were significantly predicted by skin tone, health literacy, knowledge of skin cancer factors, and perception of the supervisor's own SPBs. LDLs with skin tone that was self-perceived to be more prone to sunburns reported significantly greater use of SPBs (*β* = 0.19, *p* ≤ 0.01). Respondents with greater knowledge of skin cancer risk factors reported increased use of SPBs (*β* = 0.21, *p* ≤ 0.01). Use of greater SPBs was also higher among LDLs who had higher levels of health literacy (*β* = 0.23, *p* ≤ 0.001). The largest influence of increased SPBs was among LDLs who had greater perceptions that their supervisor used SPBs (*β* = 0.25, *p* ≤ 0.01).

## 4. Discussion

LDLs are at elevated risk for disease and injury because they are typically employed in high-risk occupations. One such risk is overexposure to UV rays. Research has established that there is a robust relationship between outdoor work and increased risk of nonmelanoma skin cancer [46]. Since SPBs are recognized as a primary prevention method to safeguard against overexposure of UV rays, the current study sought to establish what factors contribute to the use of SPBs among LDLs. Although this issue is highly significant to the work conditions experienced by LDLs, very little research has been conducted to understand this phenomenon among a group with elevated risk.

The univariate results suggest that LDLs do not adequately use SPBs, which is consistent with other studies of outdoor and Latino male workers [10, 21, 23, 24, 47]. At the univariate level, the results indicate that the least reported SPB among LDLs was use of sunscreen, and 83% reported never or sometimes using sunscreen. Given these results, there is a need to provide some type of intervention to encourage this subgroup of Latinos to adopt sun safe practices. Perhaps prevention efforts should include establishing public policies that target major construction worksites. Contractors should be mandated to establish a culture of sun safe practices by requiring them to treat sun protection as imperative as they do physical safety. One such strategy may be to ask employers who have outdoor workers to have on-site sunscreen dispensers, with at least SPF 30, to encourage sunscreen use [46]. This practice would not only help LDLs, but the all outdoor workers working at these worksites. It is more likely that LDLs would apply sunscreen if this descriptive norm is reinforced by other workers and supervisors [48]. Moreover, LDLs are likely to follow the rules and/or model the desired behavior given that they often show a great deal of respect for authority figures [49], such as their supervisors.

Consistent with the existing research, several sociodemographics, such as income, being foreign-born, skin type, and educational achievement, were significantly associated with SPBs at the bivariate level [50, 51]. However, most of these factors lost significance in the multivariate model. The only factor that remained statistically significant, after accounting for all predictors, was skin type. Consistent with other research [50], respondents who self-perceived as having more of a UV sensitive skin tone also reported the highest levels of SPBs. This phenotype risk group is not typically associated with the Latino population. However, Latinos are not a monolithic racial group; this group consists of various racial types. Many Latinos' skin tone may resemble that of non-Hispanic whites, such as some respondents who were of Mexican, Salvadoran, and Puerto Rican descent. As a result, they too exhibit some of the same risk concerns as non-Hispanic whites.

The findings that relate to health literacy and knowledge of skin cancer risk factors corroborate what other researchers have reported on Latinos: as a larger group, they do not possess a great deal of knowledge of skin cancer or its risk factors [52]. This is further exacerbated by lower levels of health literacy, lower incomes, low levels of education, and lack of access to health insurance coverage. These factors are important given that all of them, including health literacy, have been linked with poorer health outcomes [29]. Given this finding, it is imperative that prevention programming is developed to impart knowledge and awareness of skin cancer risks, as well as finding ways to increase health literacy among this at risk group. This is highly needed in light of LeBlanc and colleagues, who reported that [53], despite construction labor being a high-risk-occupation most likely to experience increased sun exposure, these workers are the least likely to have been screened for skin cancer and thus this may be associated with delayed detection and, ultimately, worse prognosis [54]. Addressing this problem is also important given that Latinos, as a group, possess some of the lowest levels of health literacy in the U.S. [32, 33] but also reported believing the most misconceptions about cancer treatment [55]. Given that few LDLs have access to preventive services, more outreach and support have to be done at the community level. For a variety of reasons, it is unlikely that LDLs will visit a physician in a formal office; however, they may be more inclined to see one in their place of employment, or some other type of community setting, such as a soccer field, park, or outdoor community event. Perhaps having mobile prevention programs that educate and raise awareness of skin cancer risks among LDLs at the worksites, or other community-based setting, may be a way to overcome this barrier.

Related, other researchers have called for the development of more culturally specific and innovative melanoma interventions to address poor sun protective practices among the Latino population [54]. However, language is something that should be considered more carefully. Although many Latinos speak Spanish, it cannot be assumed they all speak it. Many also speak other indigenous mother tongues and Spanish is a second language. In particular, some of the most recent arrivals that work as day laborers migrated from the Yucatan peninsula and Guatemala. They may speak Yucatec Maya or K'iche' Mayan, respectively. Thus, careful attention has to be given to the range of linguistic diversity that exists among LDLs.

The results of the study also point to the importance of the workplace as a barrier of SPBs. LDLs who reported perceptions that their supervisor did not practice SPBs while at work were less likely to practice sun safe practices themselves. Supervisors influence workers, formally and informally. They set the tone for what is acceptable and unacceptable behavior. In this masculine culture, workers may be stigmatized for using preventive measures [46]. This workplace culture may limit the capacity of LDLs to perform sun safe practices. This limitation is exacerbated by exploitive working condition environments, such as being denied breaks during working hours, working nonstop during peak sun times, working around the clock, and working under pressure to finish jobs quicker [46, 56, 57]. Thus, it becomes important for supervisors to either encourage sun safe practices by instituting mandatory sun practices and/or role modeling sun safe behaviors [46].

Lastly, the results highlight a need for health promotion to begin to focus more on the Latino population. More skin cancer public campaigns should be developed that target Latinos. These campaigns, however, should be developed with the understanding of the often co-occurring barriers to health care resources faced by Latinos, such as a shortage of Latino health care professionals, lacking a primary care physician, and lacking health insurance coverage [58, 59], all of which may contribute to inequitable cancer outcomes experienced by this population.

## 5. Limitations

There are some limitations to this research which should be acknowledged. This study used a nonrandom sampling design, limiting generalizability of the results and potentially introducing self-selection bias. It is plausible that LDLs opted not to participate in the study once they learned that the focus was on SPBs. Another limitation is the cross-sectional design, limiting the interpretation of any cause-and-effect relationships. In future studies, prospective design is required to examine directionality or causality. The third limitation relates to how SPBs were measured. Respondents were asked about an estimate of how often they engaged in a particular sun protective behavior. However, the measure did ask for a specific number of times they engaged in a sun safe behavior or the frequency in which they engaged in that behavior on a daily basis. Using a different measure of SPBs could have altered the results. It should also be noted that findings of this study are based solely on self-reported data. This could have introduced measurement bias in the research. Since this was a survey-based study, the potential effects of recall and social desirability biases in the findings cannot be discounted. Last, although participants were recruited from multiple locations in two different states, the study sample may not be fully representative of all LDLs. Replication of these findings in larger samples from multiple geographic areas would be beneficial.

## 6. Conclusion

It is well established that LDLs are employed in unskilled or entry-level jobs in industries with high rates of injury. Research on LDLs and SPBs is scarce, but the current study highlights inadequate SPBs reported among this high-risk population. This is significant given the strong association between outdoor work and increased risk of developing skin cancer. Thus, sun protective behaviors among LDLs are paramount. However, LDLs may face individual and structural obstacles that prevent them from fully embracing appropriate sun safe strategies. The results of this study suggest that skin type, knowledge of skin cancer risks, health literacy, and SPBs of supervisors are significant predictors of sun protective behaviors of LDLs. The results suggest that the supervisors in the workplace are the most significant influence on SPBs of LDLs. Supervisors can encourage, formally and/or informally, LDLs to consider the importance of using more SPBs given that these are malleable behaviors that could be enhanced. These results call for specific workplace policies and prevention efforts to be established that address the specific needs of this population. Doing so may create a workplace culture that embraces and encourages higher levels of SPBs among LDLs, which may lessen the heightened risk of damaged skin caused by excessive solar UVR during work hours.

## Figures and Tables

**Table 1:** Sociodemographic characteristics of the study sample (*N* = 137).

Variables and categories	Mean (standard deviation)	*n* (%)	Range
*Sun protective behaviors*	16.23 (5.11)		9–39
*Age, years*	35.40 (9.48)		18–67
*Education*			
Less than elementary school		61 (44.5%)	
Completed elementary school, but not high school		27 (19.7)	
High school diploma		2 (1.5%)	
Associate degree		41 (29.9%)	
Bachelor's degree		5 (5.6%)	
Graduate or professional degree		1 (0.08)	
*Annual income*			
Less than $20,000		95 (69.3%)	
$21,000 to $30,000		41 (29.9%)	
$31,000 to $40,000		1 (0.08)	
*Legal status*			
United States citizen		16 (11.9%)	
Naturalized citizen		12 (8.9%)	
Permanent legal resident		19 (14.1%)	
Work permit		24 (17.8%)	
Nonimmigrant visa		14 (10.3%)	
Noncitizen, nor permanent legal resident		50 (37%)	
*Number of years living in the U.S.*	11.15 (9.48)		0–45
*Health insurance coverage*			
Yes		21 (16.3%)	
No		108 (87.3%)	
*Moles on the body*			
Yes		105 (76.6%)	
No		32 (23.4%)	
*Skin type*			
Always burn, never tans		29 (21.5%)	
Usually burn, tans with difficulty		5 (3.7%)	
Sometimes mild burn, gradually tans to a light brown		24 (17.8%)	
Rarely burn, tan with ease to a moderate brown		45 (33.3%)	
Very rarely burns, tans very easily		17 (12.6%)	
Never burns, tans very easily, deeply pigmented		15 (11.1%)	
*Acculturation*	6.1 (2.9)		4–18
*Knowledge of cancer risk factors*	2.6 (1.5)		0–6
*Workplace norms *			
Supervisor supportive of SPBs	2.49 (1.13)		1–5
Supervisor supportive of SPBs	2.52 (1.08)		1–5
*Time spent outdoors at work between 10 a.m. and 4 p.m.*	4.67 (1.68)		1–7
*Perceived barriers*	44.4 (5.2)		27–59
*Self-efficacy in relation to SPBs*	32.9 (13.9)		7–70
*Health literacy*	7.93 (2.96)		3–15

*Note*. The analysis only included responses from male day laborer. One female day laborer was surveyed; however, her responses were not included in the analysis, or Table 1.

**Table 2:** Pearson's *r* zero-order correlation matrix.

Variables	(1)	(2)	(3)	(4)	(5)	(6)	(7)	(8)	(9)	(10)	(11)	(12)	(13)	(14)	(15)	(16)	(17)	(18)
(1) Sun protective behaviors	-																	
(2) Age	0.15	-																
(3) Education	0.09	0.22^*∗*^	-															
(4) Annual income	0.20^*∗*^	0.14	0.16	-														
(5) Educated in native country	−0.07	0.11	−0.23^*∗*^	−0.22^*∗*^	-													
(6) Legal Status	0.44^*∗∗*^	0.24^*∗∗*^	0.04	0.40^*∗∗*^	−0.23^*∗*^	-												
(7) Number of years living in the U.S.	0.31^*∗∗*^	0.59^*∗*^	0.05	0.24^*∗∗*^	−0.07	0.55^*∗∗*^	-											
(8) Health insurance coverage	0.02	0.21^*∗*^	0.05	0.21	−0.16	0.34^*∗*^	0.28^*∗∗*^	-										
(9) Moles on the body	0.15	0.03	−0.04	0.04	0.13	0.14	0.10	0.04	-									
(10) Skin type	0.52^*∗∗*^	0.11	0.04	0.09	0.08	0.22^*∗*^	0.06	0.07	−0.05	-								
(11) Acculturation	0.49^*∗∗*^	0.07	0.07	0.13	−0.26^*∗*^	0.74^*∗*^	0.40^*∗*^	0.25^*∗∗*^	0.05	0.33^*∗∗*^	-							
(12) Knowledge of skin cancer risk factors	0.60^*∗*^	0.16	0.04	0.03	−0.07	0.37^*∗*^	0.33^*∗∗*^	0.04	0.13	0.39^*∗∗*^	0.47^*∗∗*^	-						
(13) Support of sun protective behaviors by supervisor	0.61^*∗∗*^	0.05	0.04	0.19^*∗*^	0.01	0.18^*∗*^	0.12	0.03	0.06	0.37^*∗∗*^	0.25^*∗∗*^	0.41^*∗∗*^	-					
(14) Support of sun protective behaviors by coworkers	0.49^*∗∗*^	0.02	−0.02	0.15	0.04	0.02	0.07	0.01	0.09	0.33	0.15	0.34^*∗∗*^	0.74^*∗∗*^	-				
(15) Time spent outdoors at work between 10 a.m. & 4 p.m.	−0.01	−0.01	0.29^*∗∗*^	−0.030^*∗∗*^	0.08	−0.12	−0.12	−0.11	−0.13	0.07	−0.08	0.01	−0.01	−0.01	-			
(16) Perceived barriers	−0.18^*∗*^	−0.07	0.10	−0.11	0.12	−0.06	−0.08	−0.02	0.06	−0.06	−0.08	0.08	−0.18^*∗*^	−0.11	0.04	-		
(17) Self-efficacy in relation to SPBs	0.16	0.13	−0.18	0.25^*∗∗*^	−0.15	0.15	0.21^*∗∗*^	0.06	0.18^*∗*^	0.14	0.07	0.11	0.20^*∗*^	0.24^*∗∗*^	0.23^*∗∗*^	−0.11	-	
(18) Health literacy	0.53^*∗∗*^	0.22^*∗*^	0.16	0.03	−0.09	0.24^*∗∗*^	0.34^*∗∗*^	0.13	−0.01	0.24^*∗∗*^	0.31^*∗∗*^	0.41^*∗∗*^	0.35^*∗*^	0.25^*∗∗*^	0.21^*∗*^	−0.14	0.12	-

*Note*. ^*∗*^*p* = 0.05, ^*∗∗*^*p* = 0.01.

**Table 3:** Summary of regression analysis of variables related to sun protection behaviors.

Variables	*B*	SE_*B*_	*β*	*p* value	95% CI
Support of sun protective behaviors by supervisor	1.147	0.424	0.254	0.008	0.307–1.987
Health literacy	0.397	0.115	0.230	0.001	0.170–0.624
Knowledge of cancer risk factors	0.707	0.241	0.214	0.004	0.230–1.185
Skin type	1.979	0.668	0.191	0.004	0.654–3.304
Legal status	1.677	1.065	0.156	0.118	−0.434–3.787
Acculturation	0.131	0.158	0.075	0.409	−0.182–0.444
Support of sun protective behaviors by coworkers	0.346	0.430	0.073	0.422	−0.506–1.199
Annual income	0.544	0.672	0.051	0.420	−0.788–1.875
Barriers	−0.021	0.056	−0.021	0.712	−0.133–0.091
Number of years living in the U.S.	−0.009	0.039	−0.017	0.811	−0.086–0.067

*F*(10,109) = 20.658, *p* < 0.001, *R*^2^ (adjusted *R*^2^) = 0.655 (0.623); dependent variable is sun protection behavior; *B* = unstandardized coefficient; SE_*B*_ = standard error of the coefficient; *β* = standardized coefficient; *p* = level of significance; and CI = confidence interval.
